# Advanced maternal age compromises fetal growth and induces sex-specific changes in placental phenotype in rats

**DOI:** 10.1038/s41598-019-53199-x

**Published:** 2019-11-28

**Authors:** Tina Napso, Yin-Po Hung, Sandra T. Davidge, Alison S. Care, Amanda N. Sferruzzi-Perri

**Affiliations:** 1 Centre for Trophoblast Research, Department of Physiology, Development and Neuroscience, University of Cambridge, Cambridge, UK; 2 Department of Obstetrics and Gynaecology, Women and Children’s Health Research Institute, University of Alberta, Edmonton, Alberta Canada; 3 Robinson Research Institute and Adelaide Medical School, University of Adelaide, South Australia, Australia

**Keywords:** Developmental biology, Reproductive biology

## Abstract

Advanced maternal age is associated with an increased risk of pregnancy complications. It programmes sex-specific cardiovascular dysfunction in rat offspring, however the intrauterine mechanisms involved remain unknown. This study in the rat assessed the impact of advanced maternal age on placental phenotype in relation to the growth of female and male fetuses. We show that relative to young (3–4 months) dams, advanced maternal age (9.5–10 months) compromises growth of both female and male fetuses but affects the placental phenotype sex-specifically. In placentas from aged versus young dams, the size of the placental transport and endocrine zones were increased and expression of *Igf2* (+41%) and placental lactogen (*Prl3b1*: +59%) genes were upregulated in female, but not male fetuses. Placental abundance of IGF2 protein also decreased in the placenta of males only (−95%). Moreover, in placentas from aged versus young dams, glucocorticoid metabolism (*11β-hsd2*: +63% and *11β-hsd1*: −33%) was higher in females, but lower in males (*11β-hsd2*: −50% and *11β-hsd1*: unaltered). There was however, no change in the placental abundance of 11β-HSD2 protein in aged versus young dams regardless of fetal sex. Levels of oxidative stress in the placenta were increased in female and male fetuses (+57% and +90%, respectively) and apoptosis increased specifically in the placenta of males from aged rat dams (+700%). Thus, advanced maternal age alters placental phenotype in a sex-specific fashion. These sexually-divergent changes may play a role in determining health outcomes of female and male offspring of aged mothers.

## Introduction

Pregnancy at an advanced maternal age (≥35 years of age) is increasing, particularly in developed countries. For instance, in the USA, Canada, UK and Australia, births to women 35 years and older constitute 14–22% of total live births^1,2^. Birth at an advanced maternal age involves higher risks for both mothers and babies, including an increased risk of developing complications such as gestational diabetes, placenta praevia, gestational hypertension, stillbirth and caesarean section delivery^3,4^. Furthermore, advanced maternal age is an independent risk factor for the development of preeclampsia^5^. Epidemiological data demonstrate that infants born to older mothers are more likely to be born preterm or small for gestational age^6^. Moreover, there are higher levels of fetal congenital anomalies and chromosomal abnormalities^7^.

Advanced maternal age can also affect the intrauterine environment, with previous studies showing an impaired decidual reaction and reduced uterine prostaglandin synthesis^8^, as well as increased loss during the peri-implantation period^9^. There are also changes in the microstructure of the uterine luminal epithelium, particularly in the microvillous architecture^8,10^. These changes may affect the ability of the blastocyst to attach and of trophoblast cells to invade into the underlying decidua^8^. In addition, impaired oocyte developmental potential and a suboptimal intrauterine environment contribute to reduced embryo developmental competence, all of which have been described in advanced maternal age^8,11^. Aging also affects the immune cell population in the decidua and the uterine response to hormones that invoke decidualisation in the mouse and lead to impaired embryonic and placental development^12^. These effects appear to be independent of the oocyte and embryo, as they could be largely rescued by transferring mouse embryos from older dams to young dams^12^.

We have previously described that placental weight is increased in advanced maternal age rats, which is consistent with other studies in advanced maternal age mice^13^ and women^3–6,14–16^. There is substantial evidence in other rodent models to suggest that placental structure, transport and endocrine function, are regulated by the maternal environment and that adverse conditions such as maternal over or under-nutrition, micro and macronutrient deficiency, obesity and hypoxia alter these sensitive processes^17^. Moreover, in many of these conditions, there are alterations in the ability of the placenta to protect the fetus from circulating maternal glucocorticoids, which in turn, can profoundly affect fetal development and later offspring health^18–20^. However, limited information is available on how placental structure and function is altered by advanced maternal age.

In women of advanced maternal age, placental transport capacity is increased^16^. In a study that assessed placental function in mice, the amino acid transport capacity in placentae from advanced maternal age mice (^14^C-methyl amino isobutyric acid and ^3^H-taurine clearance) is decreased^13^. Furthermore, advanced maternal age has been shown to alter the main direction of placentation and the trophoblast compartment in the mouse^12^. It is becoming increasingly evident that fetal sex influences placental and offspring outcomes in response to adverse maternal environments during gestation^21,22^. However, little information is available about whether advanced maternal age leads to any sex-specific changes in placental structure and functional capacity. Such sex-specific changes in placental phenotype may be linked to the early-life programming of cardiovascular disease susceptibility in offspring born of aged dams, which we have shown to be sex-dependent^23,24^.

We hypothesized that the reduced fetal weight and impaired pregnancy success observed in our rat model of advanced maternal age, may be due to sex-specific alterations in placental morphological development and nutrient transport function. Here we show that advanced maternal age affects placental development and functional capacity in a sex-dependant fashion in the rat.

## Results

### Advanced age affects maternal metabolic phenotype during pregnancy

Young (3–4 months old) or aged (9.5–10 months old) Sprague Dawley female rats were time-mated with young male Sprague Dawley rats (3–5 months old) and the impact of advanced maternal age on maternal metabolic physiology and conceptus development were evaluated on gestational day (GD) 20. In a previously published study using this cohort, we showed that aged dams had greater fat percentage and gross body weight compared to young dams, together with changes in uterine artery function in late pregnancy^25^. To further indicate maternal metabolic state, in this study, we evaluated serum metabolite and hormone concentrations in aged versus young dams. Glucose concentration in the maternal serum was elevated by 11% in aged dams compared to young (p < 0.05, Table 1). However, serum insulin, leptin, cholesterol and free fatty acid concentrations were not altered by advanced maternal age (Table 1).

**Table 1:** Serum hormone and metabolite concentrations in young and aged dams.

	Young	Aged	t-test
Insulin (ug/L)	7.39 ± 2.9	3.76 ± 0.5	p = 0.25
Leptin (pg/ml)	2455.8 ± 467	5367 ± 1670	p = 0.124
Glucose (mmol/l)	6.97 ± 0.06	**7.73** ± 0.29*	p = 0.04
Cholesterol (mmol/l)	1.95 ± 0.09	1.75 ± 0.11	p = 0.2
FFA (umol/l)	1211.1 ± 267	1098.8 ± 95	p = 0.7

Data presented as mean ± SEM. Data are from young or aged females (n = 6 females per group). *Significant difference between groups by unpaired t test (p < 0.05).

### Advanced maternal age reduces fetal weight and placenta efficiency

**Figure 1:**
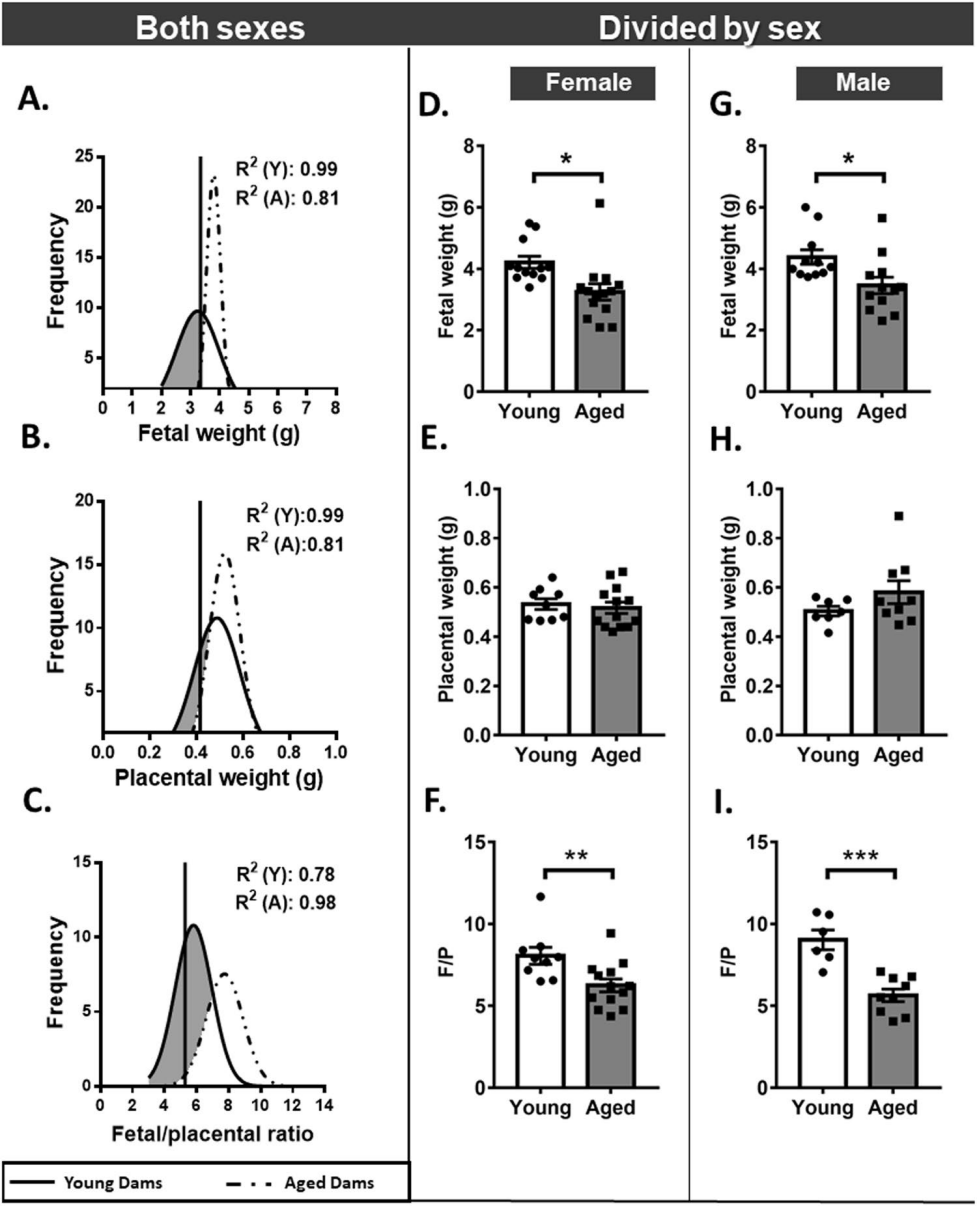
Fetal and placental weights in young versus aged dams. Distribution curves for fetal weight (**A**), placental weight (**B**) and placental efficiency (**C**) shown independent of fetal sex. Vertical solid line represents the 5th centile of the young curve (3.345 g, 0.416 g and 5.28 in A, B and C, respectively). Fetal weight (**D**,**G**), placental weight (**F,H**) and placental efficiency (**E**,**I**) for female and male fetuses, respectively. Data displayed as mean + SEM and are from 9–20 fetuses and placentas from 9–12 dams per group (1–2 conceptuses per litter used). Statistical difference between young and aged dams was determined by unpaired student t test, *p < 0.05, **p < 0.01 ***p < 0.001.

We have previously reported that in aged dams, litter size was reduced and greater fetal loss was observed compared to young dams^25^. Here we show that in the subset of dams assessed in this study, weight distribution curves for viable fetuses are shifted to the left towards the lower fetal weight range in aged dams (Fig. 1A). Indeed, more than half of the fetuses (55%) from aged dams displayed a weight below the 5th centile for young dams (Fig. 1A).When accounting for fetal sex, average weight of both female and male fetuses was reduced by ~20–25% in aged dams compared to young dams (Fig. 1D,G). The reduction in fetal weight in aged dams was associated with significant decrease in the absolute weight of heart, brain and liver in males (p < 0.0 5) but not in females (Table 2). There was however, no effect of maternal age on the relative weight of any fetal organ analysed in male or females, suggesting that fetuses were symmetrically growth restricted (Table 2). Advanced maternal age did not affect placental weight or placental weight distribution (Fig. 1B,E,H). However, there was an overall leftward shift in placental efficiency distribution (estimated as the ratio of fetal weight to placental weight) (Fig. 1C) and placental efficiency was reduced by 22% for females and 37% for male fetuses from aged versus young dams (p < 0.01 and p < 0.001, respectively; Fig. 1F,I). Thus, advanced maternal age compromises fetal development and placental efficiency.

**Table 2:** Fetal organ weights in young versus aged dams.

	Female		Male	
	Young	Aged	Young	Aged
Heart (g)	0.025 ± 0.001	0.022 ± 0.0008	0.026 ± 0.0008	**0.022 **± 0.0007^*^
Heart (% of Fw)	0.669 ± 0.04	0.660 ± 0.03	0.650 ± 0.02	0.641 ± 0.03
Brain (g)	0.186 ± 0.003	0.176 ± 0.002	0.193 ± 0.002	**0.174 **± 0.004^*^
Brain (% of Fw)	4.9 ± 0.07	5.2 ± 0.18	4.8 ± 0.09	5.0 ± 0.19
Liver (g)	0.331 ± 0.007	0.356 ± 0.011	0.308 ± 0.016	**0.310 **± 0.016^*^
Liver (% of Fw)	8.71 ± 0.20	9.16 ± 0.56	8.94 ± 0.19	8.96 ± 0.26
Kidney (g)	0.028 ± 0.002	0.031 ± 0.0009	0.026 ± 0.0007	0.029 ± 0.001
Kidney (% of Fw)	0.73 ± 0.05	0.77 ± 0.02	0.80 ± 0.02	0.84 ± 0.02
Brain/Liver	0.56 ± 0.016	0.54 ± 0.016	0.58 ± 0.029	0.56 ± 0.023

Data presented as mean ± SEM. Data are from 7 fetuses per sex from 4–5 dams per group. Fw: fetal weight. Significant difference for maternal age within one sex was determined by unpaired t test, *p < 0.05.

### Advanced maternal age alters placental structure

**Figure 2:**
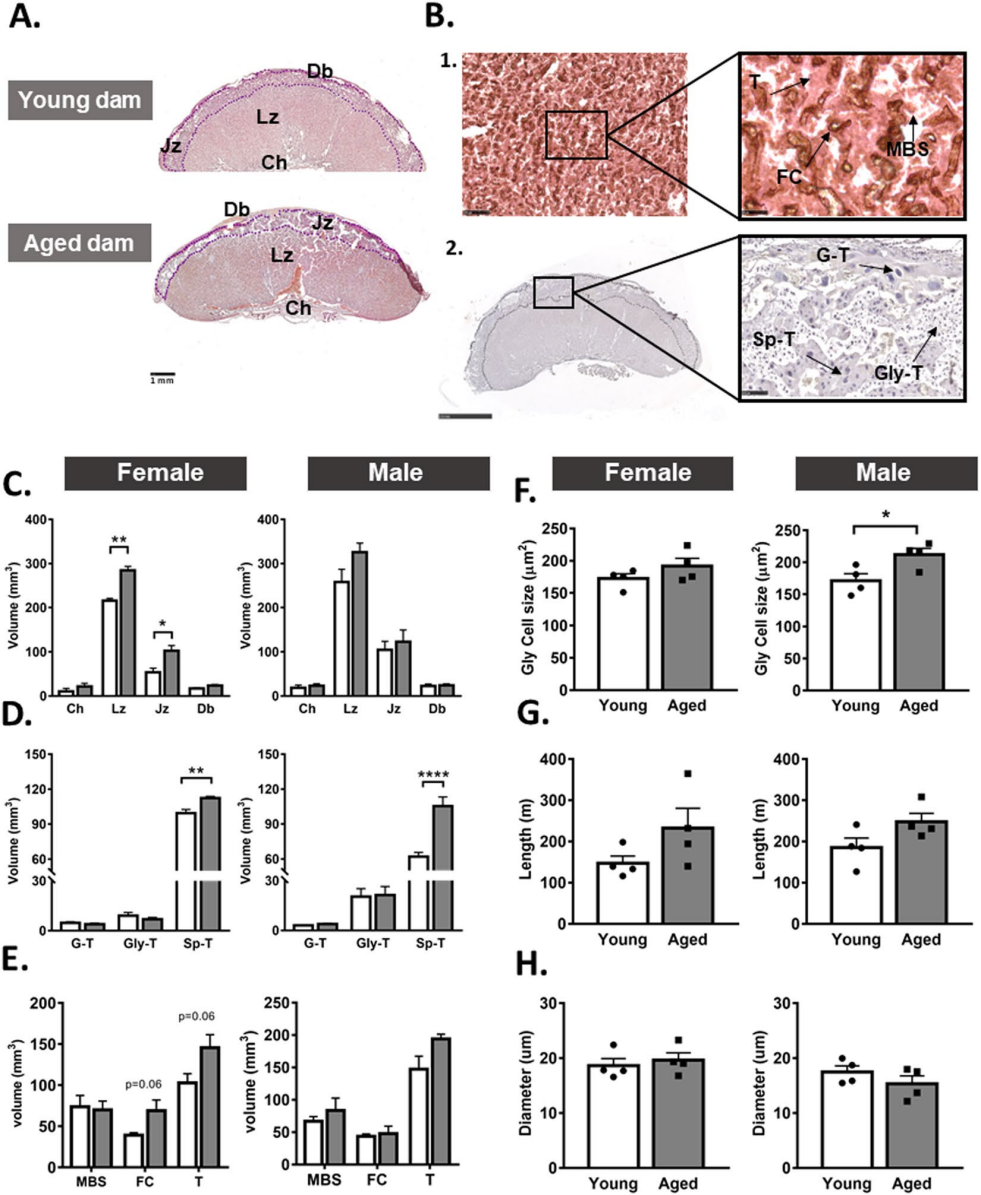
Placental morphology in young versus aged dams. Representative images of haematoxylin and eosin stained placentas (**A**). Representative images of placentas showing the low and high magnification structure of the labyrinthine zone by immuno-staining for laminin and cytokeratin (**B-1**), as well as the structure of the junctional zone by staining with haematoxylin and eosin (**B-2**). Placental region volumes (**C**), junctional zone cell volumes (**D**), labyrinthine zone compartment volumes (**E**), glycogen cell size (**F**) and labyrinthine fetal vessel length (**G**) and diameter (**H**) in females and males. Data are from 4 placentas per group, each from different litter and presented as mean + SEM values. Significant difference between young and aged dams were determined by unpaired student t test, *p < 0.05. Ch: chorion, Db: decidua, FC: fetal capillaries, G-T: giant cells, Gly-T: glycogen cells, Jz: junctional zone, Lz: labyrinthine zone, MBS: maternal blood spaces, Sp-T: spongiotrophoblast, Troph: trophoblast.

To determine whether the reduction in placental efficiency in aged dams may be related to morphological changes to the placenta, we analysed placental structure from female and male fetuses using stereology (Fig. 2A,B). The estimated volumes of the placental endocrine junctional zone (Jz) and transport labyrinthine zone (Lz) were increased for female, but not male fetuses in aged versus young dams (p < 0.05; Fig. 2C). The volumes of the maternal decidua and the placental chorion were not affected by maternal age in either female or male fetuses.

In the Jz, the volume of the spongiotrophoblast cells (Sp-T) was increased by 13% in females and 70% in male fetuses of aged compared to young dams (p < 0.001; Fig. 2D). The volume of glycogen cells (GlyT) and giant cells (G-T) in the placental Jz were not altered by maternal age in either female or male fetuses. However, in male fetuses but not females, the cell size of the Gly-T was significantly increased in aged dams compared to male placentas from young dams (p < 0.05, Fig. 2F).

In the Lz of female, but not male fetuses, the estimated volumes of fetal capillary (FC) and trophoblast trended towards an increase in aged versus young dams (both p = 0.06; Fig. 2E). In both female and male fetuses, the volumes of maternal blood spaces and trophoblast in the Lz were similar between aged and young dams (Fig. 2E). There was no effect of maternal age on FC length and diameter (Fig. 2G,H), thickness of trophoblast barrier to diffusion, surface area for exchange or the theoretical capacity for diffusion of molecules like oxygen (Table 3) in either female or male pups in aged versus young dams. Taken together, these data indicate that advanced maternal age affects placental structure in a sex-dependant manner. However, the changes in placental structure do not appear to completely explain the reduction in placental efficiency observed in aged dams.

**Table 3:** Placental exchange characteristics in young versus aged dams.

	Female		Male	
	Young	Aged	Young	Aged
**Barrier thickness (µm)**				
	3.48 ± 0.21	3.17 ± 0.40	3.09 ± 0.14	3.82 ± 0.35
**Surface area (cm**^**2**^**)**				
**MBS**	4.1 ± 0.71	3.38 ± 0.51	3.24 ± 0.34	4.06 ± 0.90
**FC**	2.21 ± 0.26	3.34 ± 0.62	2.10 ± 0.18	2.31 ± 0.55
**TDC (mm**^**2**^**/min/kPa)**				
	0.00118 ± 0.00021	0.00134 ± 0.00025	0.00115 ± 0.00013	0.00119 ± 0.00035

Data are from 4 placentas each from a different litter per group and are presented as mean ± SEM. No significance was detected between young and aged dams by unpaired t-test. TDC: total diffusing capacity.

### Advanced maternal age changes placental gene and protein expression in both female and male fetuses

**Figure 3:**
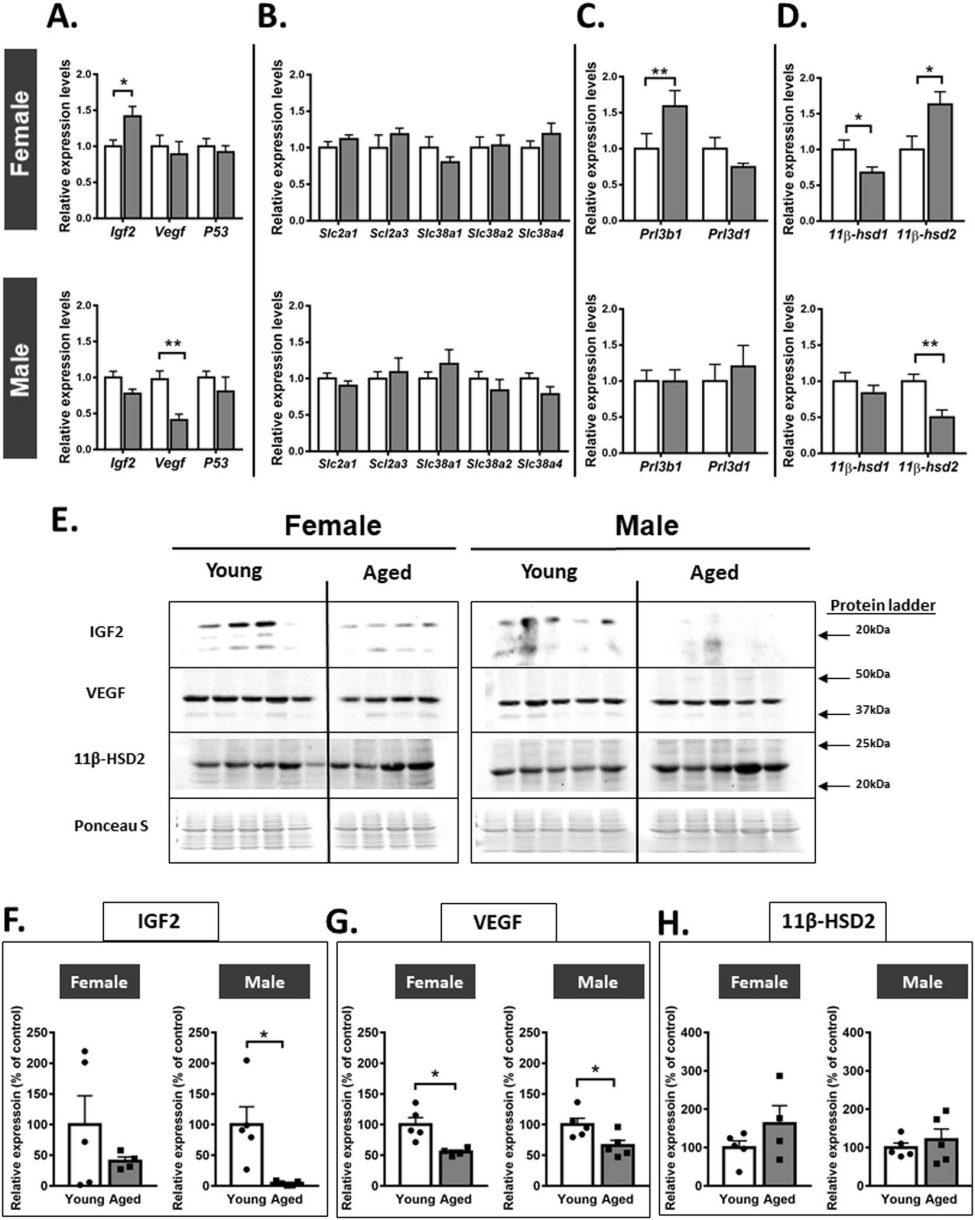
Placental gene and protein expression in young versus aged dams. Expression of growth regulatory (**A**), nutrient transporter (**B**), placental lactogen (**C**) and glucocorticoid metabolism (**D**) genes in placentas from female and male fetuses. Western blot images of IGF2, VEGF and 11-βHSD2 (**E**) abundance in female and male placentas with quantification of proteins adjusted for Ponceau S staining (**F–H**, respectively). For gene expression analyses, data are from 10–12 placentas from 5 litters per group and for protein abundance, data are from 4–5 placentas each from different litter. All data are presented as mean + SEM values. Significant difference between young and aged dams were determined by unpaired student t test, *p < 0.05.

To further examine the mechanisms underlying impaired placental efficiency in aged dams, we quantified the placental expression of growth-regulatory genes (*Igf2*, *Vegf* and *p53)*, system A amino acid (*Slc38a*) transporters, glucose transporters (*Slc2a*), hormones (placental lactogens 1 and 2: *Prl3d1* and *Prl3b1*) and enzymes which metabolise and control glucocorticoid actions in the conceptus (*11β-hsd1* and *11β-hsd2*, which increase and reduce glucocorticoid activity, respectively). In female, but not male fetuses, placental mRNA expression of *Igf2, Prl3b1* and *11β-hsd2* were increased, whereas *11β-hsd1* was reduced in aged compared to young dams (p < 0.05; Fig. 3A–D). In male fetuses, advanced maternal age reduced placental *Vegf* and *11β-hsd2* expression without affecting the expression of other genes assessed (p < 0.05; Fig. 3A–D).

In males, the abundance of IGF2 protein in the placenta was significantly reduced, but unaltered in females by advanced maternal age (Fig. 3F). VEGF abundance was reduced by ~50% in the placenta of both female and male fetuses of aged dams (Fig. 3G). However, the placenta abundance of 11β-HSD2 protein tended to be elevated in females (p = 0.19) and not altered in the placenta of males from aged dams (Fig. 3H). These data demonstrate that advanced maternal age affects the expression of genes and proteins that control the growth and function of the placenta in a manner that depends partially on fetal sex.

### Advanced maternal age is associated with increased levels of placental oxidative stress and apoptosis in male but not female fetuses

**Figure 4:**
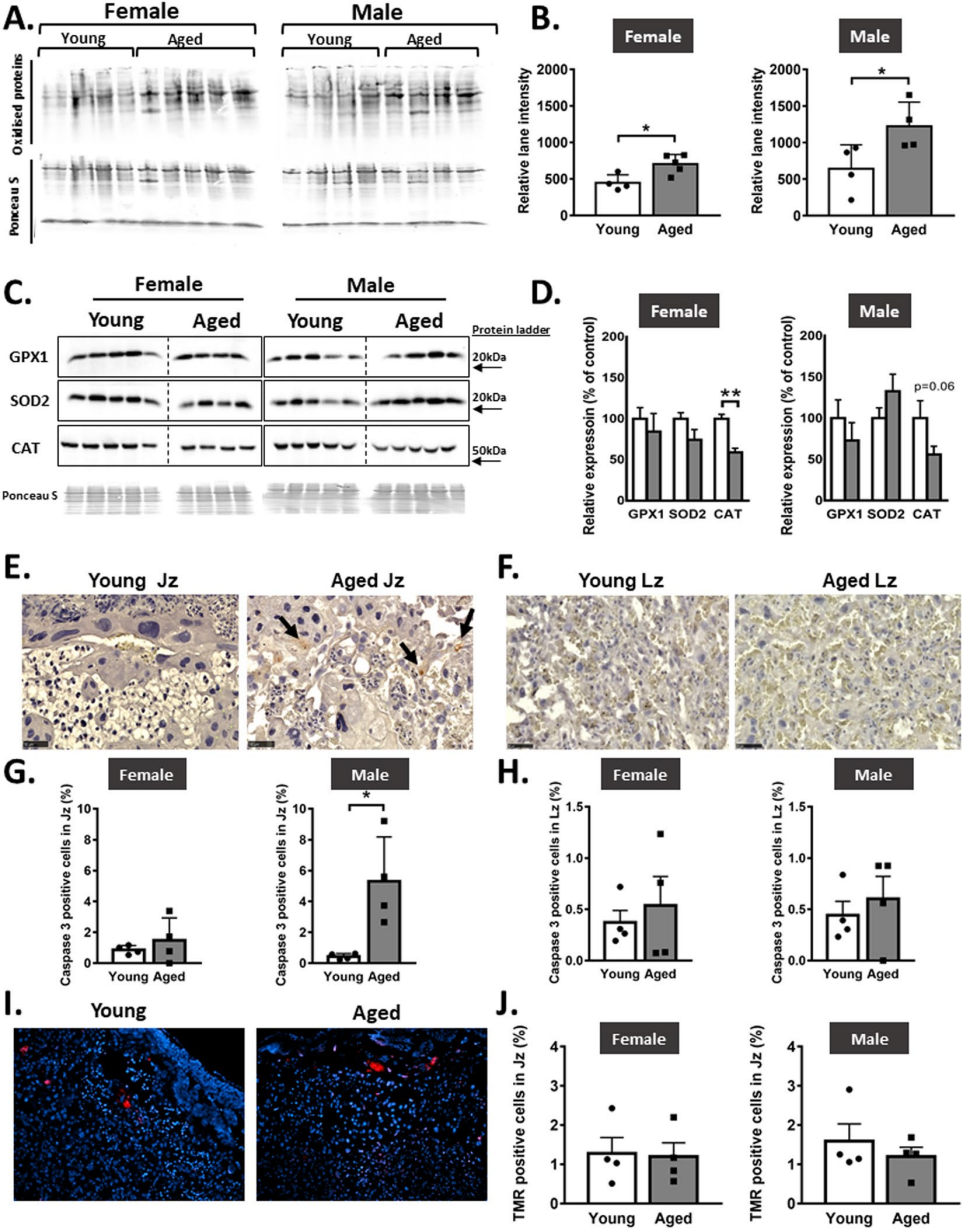
Placental oxidative stress and apoptosis in young versus aged dams. Images of entire immunoblots showing oxidatively damaged proteins (**A**) with protein quantitation (**B**) in females and males. Western blot images of antioxidant enzymes GPX1, SOD2 and CAT (**C**) in female and male placentas with abundance of proteins adjusted for Ponceau S staining (**D**). Representative images of sections showing cleaved caspase 3 immunohistochemistry in the junctional zone (**E**) and labyrinthine zone (**F**) with abundance quantified (**G**,**H**, respectively) in female and male placentas. Representative images of sections showing Tunel staining (**I**) with abundance quantified in the junctional zone (**J**) in female and male placentas. Data are from n = 4 placentas per group, each from different litter and presented as mean + SEM values. Jz: junctional zone, Lz: labyrinth zone. Significant difference between young and aged dams were determined by unpaired student t test, *p < 0.05, **p < 0.01.

Elevated levels of oxidative stress have been reported in the placenta of compromised human pregnancies and in experimental animals exposed to adverse gestational environments^26–28^. It can result from an imbalance between prooxidant and antioxidant systems and lead to apoptosis. Hence, the abundance of oxidative stress, antioxidant enzymes and apoptosis was assessed in the placenta of female and male fetuses of aged versus young dams. In aged compared to young dams, the level of protein carbonylation, a marker of oxidative stress was increased by ~57% and ~90% in the placenta of female and male fetuses, respectively (p < 0.05; Fig. 4A,B). The abundance of antioxidant enzymes, anti-glutathione peroxidase 1 (GPX1) and superoxide dismutase-2 (SOD2) in the placenta of either female or male pups were not different with maternal age. In placentas from female fetuses, the abundance of another antioxidant, catalase (CAT) was reduced by 30% (p < 0.01) whereas only a trend towards a reduction was observed in males (p < 0.06) (Fig. 4C,D). The abundance of cleaved caspase, an indicator of apoptosis was elevated in the placental Jz of male, but not female fetuses from aged dams (p < 0.05; Fig. 4E,G). The abundance of cleaved caspase in the placental Lz was not affected by maternal age in either female or male fetuses (Fig. 4F,H). Activation of the end stage of apoptosis, indicated by TMR red staining in the placental Jz was not significantly different with maternal age, regardless of sex (Fig. 4I,J). There was no TMR red staining detected in the placenta Lz. Thus, advanced maternal age increases oxidative stress in the placenta of female and male fetuses, with placentas only from males showing elevated levels of apoptosis in the Jz.

## Discussion

This study demonstrates that advanced maternal age modifies placental phenotype and hence its ability to support fetal growth. In particular, it affects placental morphological development and expression of genes and proteins fundamentally important in placental growth, nutrient transfer, endocrine control of maternal physiology and control of fetal glucocorticoid exposure. Advanced maternal age induces oxidative stress and cell death in the placenta, in a partially sex-dependent manner. Moreover, gene expression changes in the placentas of female fetuses were largely beneficial, with an upregulation of genes that support placental function. However, gene expression changes in placentae of male fetuses were generally detrimental for placental growth and functional phenotype in aged dams. Both female and male fetuses were similarly growth restricted, although absolute weight of male fetal heart, brain and liver were reduced in aged dams versus young dams. Moreover, our previous studies have shown poorer cardiovascular outcomes for adult male offspring from aged dams^24^. Taken together, our data demonstrate that the effects of advanced maternal age on fetal growth and also later-life offspring health may be mediated, at least in part, by sexually-dimorphic changes in the placenta during pregnancy. These findings may have relevance for developing targeted interventions to improve placental development and function and thus the fetal growth and development trajectory for mothers of advanced maternal age.

Placental development and expression of genes and proteins that impact the function of the endocrine Jz in the placenta was altered in aged dams. The spongiotrophoblast compartment was larger in the placenta of both female and male fetuses of aged dams, whereas the glycogen cells were significantly larger in size only in the male placenta of the aged dams, the latter of which may reflect a more advanced Jz phenotype^29^. These Jz changes would be expected to have enhanced the placental capacity to secrete hormones^30^ as well as potentially provided a larger supply of stored glucose (as glycogen) for generating energy for the final phase of fetal growth^31^ in aged dams. However, in male fetuses of aged dams there were reduced IGF2 abundance and elevated expression of apoptosis markers in the Jz, particularly in the spongiotrophoblast, which may have compromised the functional capacity of the Jz, even if they were in greater volume. In females, but not males, placental expression of *Prl3b1*, which encodes placental lactogen 2 was increased in line with spongiotrophoblast expansion^32^. This finding is consistent with work in mice which found enhanced expression of placental lactogen genes in aged dams, although sex-related effects were not investigated^12^. Placental hormones such as placental lactogen and IGF2, change pancreatic β-cell function and reduce insulin sensitivity and glucose utilisation in the mother^33–35^. Changes in glucose handling of the placenta may be exacerbated by, or related to, the elevated circulating glucose concentration observed in our aged rat dams. Future studies are needed to determine the interaction between an altered maternal metabolic state and changes in the endocrine placental Jz during gestation. This may enhance our understanding of why pregnancy at an advanced maternal age is associated with an increased risk of gestational diabetes in women^4^. Taken together, our data suggest that endocrine function of placentas from female fetuses, and to a lesser extent, placentas from male fetuses, adapt in response to the altered gestational environment in aged compared to young rat dams. Our data are consistent with other reports that demonstrate beneficial changes in placental endocrine phenotype with other adverse gestational environments that compromise the ability of the mother to support conceptus development in rodents^36–40^.

The volume of the Lz was significantly increased in placentas from female fetuses from aged dams. However, placental expression of amino acid transporters was unchanged in both female and male fetuses in aged, compared to young dams. Furthermore, in both female and male fetuses, placental expression of glucose transporters (*Slc2a1* and *Slc2a3*) was not compromised by advanced maternal age, unlike other adverse maternal conditions in rodents and women, such as excess glucocorticoids, malnutrition, obesity, hyperglycemia and hypoxia^41–47^. Interestingly, placental abundance of VEGF was diminished in both female and male pups from aged dams. Changes in VEGF expression with maternal age may affect vascular branching in the placental Lz, with downstream consequences for blood supply and oxidation levels in the placenta, as well as fetal growth^48^. Indeed, advanced maternal age increased levels of oxidative stress in the placenta in both sexes. The reduced VEGF and increased oxidative stress in the placenta are consistent with the altered uterine and umbilical artery function that may diminish the supply of blood to the placenta and fetus in aged rat dams in late pregnancy^25^. However, the levels of oxidative stress in the placenta were increased to a greater extent in male fetuses when compared to female fetuses in aged dams. This was despite the observation that placentas from female but not male fetuses showed reduced abundance of the antioxidant protein, catalase/CAT in aged dams (there was no effect of maternal age on the abundance of GPX1 or SOD2 protein). Our observation are in line with previous studies showing increased oxidative stress in placentas from aged dams resulting in fetal growth restriction^13,49^.

Changes in the endocrine and transport phenotype in the placenta of female fetuses in aged rats may be partly mediated by the altered expression of the IGF2. The *Igf2* gene is important for the differentiation of glycogen cells and expression placental lactogen 2 in the rodent placenta^50–52^. It also promotes the morphogenesis of the Lz and regulates the expression of System A amino acid transporters, including *Slc38a4* in mice^53,54^. Studies in mice, rats, guinea pigs and non-human primates have shown that expression of *Igf2* correlates with phenotypic changes in the placenta in response an altered maternal environment (reviewed in^35^). Furthermore, previous work has shown that placental *Igf2* is essential for the placenta to morphologically and functionally adapt to maternal undernutrition in mice^55^. In the current study, the expression of the *Igf2* gene was increased in female, but unchanged male placentas of aged dams. This is in line with previous work showing that placental *Igf2* transcript expression is more responsive in females than males when the gestational environment is unfavourable^39,43,56^. However, in the current study, placental IGF2 protein abundance was unchanged in females and reduced in males in response to advanced maternal age. The mechanisms underlying the sexually-disparate response and discrepancy between the gene and protein expression of *Igf2*/IGF2 in the placenta of aged dams require further investigation. Furthermore, additional work is required to define the contribution of changes in placental *Igf2*/IGF2 to the morphological and functional phenotype of the placenta from female and male fetuses of aged dams.

Phenotypic responses of the placenta in aged rats may also relate to changes in the placental handling of maternal glucocorticoids. Previous studies have shown that increased exposure to glucocorticoids compromises placental development^57,58^ and induces apoptosis specifically in the Jz^59^. It also perturbs *Vegf*^60^, placental lactogen, *Igf2*^61^ and system A amino acid transporter expression in the rodent placenta^57,58,62^. Moreover, antenatal glucocorticoids induce oxidative stress in the human placenta of male infants^63^. The disparity of the placental response to advanced maternal age with fetal sex may therefore relate to differences in the expression of *11β-hsd2* by the placenta in female and males of young rats seen herein. Most notably, they may also be linked to the observation that in female fetuses of aged dams, increased *11β-hsd2* and decreased *11β-hsd1* expression by the placenta would have limited feto-placental exposure to maternal glucocorticoids, whereas in male fetuses, reduced placental *11β-hsd2* and maintained *11β-hsd1* expression would have increased glucocorticoid exposure of the conceptus. Previous work in sheep, pigs and rodents have shown that other adverse maternal environments, including malnutrition, obesity, stress and hypoxia reduce placental *11β-hsd2* expression^42,64–67^, however only few have considered the influence of fetal sex^43,68,69^.

The sex-dependent changes in placental glucocorticoid handing induced by advanced maternal age may also explain offspring outcomes. Work in small and large animal models has demonstrated that elevated prenatal exposure to glucocorticoids affects fetal development and permanently alters organ structure and function, predisposing to diseases, such as hypertension in the offspring in later life^18–20^. Indeed, glucocorticoids can affect cardiomyocyte differentiation and induce oxidative stress in tissues including the heart and vasculature^70^. Even though fetal weight was similarly compromised for female and male fetuses from aged dams and both sexes experience postnatal catch up growth, we have previously found that adult male offspring of aged dams have a greater propensity to develop cardiovascular dysfunction as adults, when compared to female offspring^23,24^. Therefore, possible increases in glucocorticoid exposure of the male fetus, via decreased placental *11β-hsd2* may have contributed to the programming of cardiovascular dysfunction seen specifically in male offspring of aged dams. However, caution is warranted when interpreting changes in placental glucocorticoid handing during gestation in aged rat dams. In particular, and despite changes in gene expression, placental 11β-HSD2 abundance only tended to be elevated (non-significant) in females and was unchanged in males of aged dams. Therefore, further work is required to assess more directly the role of placental glucocorticoid handling in placenta phenotype and offspring outcome of females and males from aged dams.

The current study applied morphological and molecular (gene and protein expression) approaches to assess phenotypic changes in the placenta of female and male fetuses in response to advanced maternal age in the rat. However, further studies assessing the transport, endocrine and barrier functions of the placenta using *in vivo* and isolated organ approaches in aged dams are warranted. In the current study, only one gestational timepoint (GD20) was assessed in our rat dams. Although others have shown that there are morphological and transcriptional changes in the placenta of aged dams compared to young dams earlier in gestation, on GD11.5 in the mouse^12^. Thus, it is likely that some changes in placental phenotype with maternal age in our rat dams may emerge much earlier in gestation and hence, a time-course of the phenotypic changes in the placenta and fetal growth with advanced maternal age are needed in future work. In rodents, direct conclusions between placental traits and offspring outcome are challenging. Thus, there is much to be gained by analysing placental phenotype with subsequent follow-up of the child in women of advanced maternal age.

**Figure 5:**
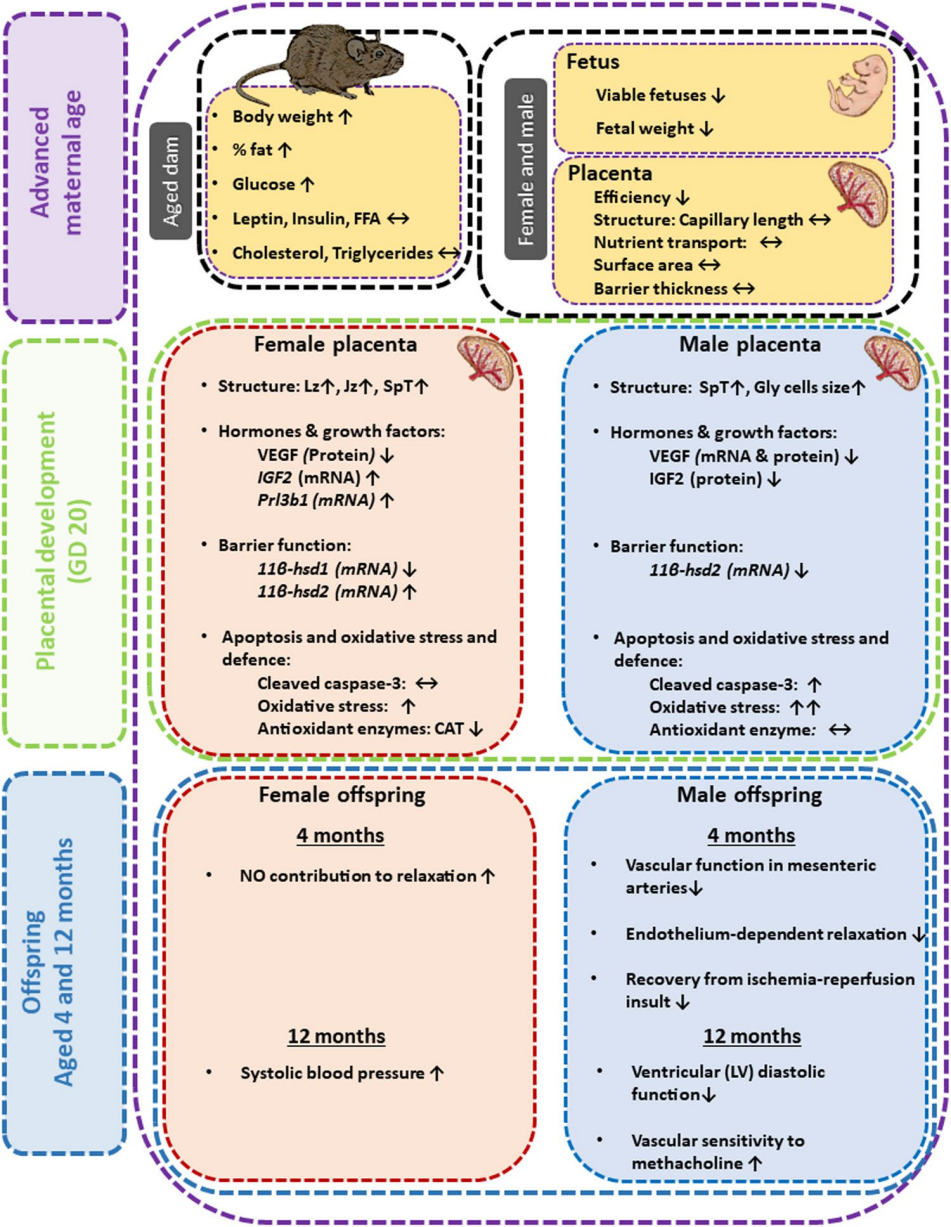
Summary illustration showing the effects of maternal age on the placenta, fetus and offspring (at 4 month and 12 months of age as published in Cooke *et al*.^23^ and Shah *et al*.^24^) for females and males. Gly-T: glycogen cells, Jz: junctional zone, Lz: labyrinthine zone, Sp-T: spongiotrophoblast, Troph: trophoblast.

In summary, advanced maternal age affects the phenotype of the rat placenta in a sex-dependent manner (Fig. 5). In female fetuses from aged dams, there were largely beneficial changes in structure and/or expression of genes related to the function of both the placental transport and endocrine zone, which would aid materno-fetal nutrient transfer and were related to the up-regulation of *Igf2* and improved glucocorticoid handling in the placenta of aged dams, compared to young dams. Whereas, in male fetuses of aged rats, there were no beneficial changes in placental transport zone formation or hormone expression and reduced IGF2 when compared to placentas from young dams. There also appeared to be diminished protection of the conceptus from glucocorticoids, greater induction of oxidative stress (compared with female even though the placenta of females had reduced CAT abundance), and elevated levels of apoptosis in the male placenta of aged rats. The maternal and sex-dependent fetal signals driving changes in placenta phenotype in aged dams require elucidation. However, the sexually-divergent changes in placental responses to the environment provided by an aged mother, likely play a central role in determining health outcomes of female and male offspring^23,24^. These results have implications for the management human pregnancies, particularly in developed countries where women are more often entering pregnancy at an older age.

## Methods

### Animal ethics and aged model

Rat experiments were conducted under the Canadian Council on Animal Care guidelines and were approved by the University of Alberta Health Sciences Animal Policy and Welfare Committee. All experimental protocols conformed to the National Institutes of Health’s Guide for the Care and Use of Laboratory Animals (eighth edition, revised 2011).

Sprague Dawley female rats (3 months of age) were purchased from Charles River (St. Constant, QC) and housed in a temperature-controlled room with a 10:14 h light: dark cycle. Female rats were randomly allocated to the young (3–4 months old) or aged (9.5–10 months old) group, corresponding to approximately 35 years of age in humans (i.e. defined for humans as advanced maternal age) when considering such milestones as weaning, sexual maturity, skeletal maturity and reproductive senescence^69^, and were mated with young males (3–5 months old). To control for differences in body weight, dams for the aged group were kept on a controlled-feeding regime until pregnancy. Young rats were fed ad libitum throughout the study. Aged rats were maintained on a controlled feeding regime from 3 months of age until pregnancy, when they were also fed ad libitum. In the controlled feeding regime, rats were fed 6 pellets of chow per rat per day, based on the National Research Council recommendations^71^. As rats were housed in pairs, they were weighed weekly to ensure they did not lose weight. Pairs were rotated if this occurred. The presence of sperm in a vaginal smear indicated mating had occurred and was designated as GD 0 out of 22 days of rat pregnancy. At GD 20, dams were anesthetized with isoflurane and killed by cardiac exsanguination. Maternal blood was kept for serum measurement of metabolite and hormone concentrations. Litter size, number of viable and resorbing fetuses, and placental, fetuses and fetal organs weights were recorded. Placentas were bisected and fixed in paraformaldehyde for morphological assessment or snap frozen for molecular analysis. Frozen placentas were pulverized for RNA, DNA and protein extraction.

### Fetal sex determination

Due to time and technical constraints at the post-mortems, many, but not all the conceptuses within each litter were collected for sex determination. Fetal sex was determined using extracted placental DNA and PCR amplification of the *Sry* (primers: 5′-TACAGCCTGAGGACATATTA-3′ and 5′-GCACTTTAACCCTTCGATGA-3′; product size: 317 bp) and *Actin* (primers: 5′-AGCCATGTACGTAGCCATCC-3′ and 5′-TGTGGTGGTGAAGCTGTAGC-3′; product size: 220 bp). PCR products were visualised by electrophoresis using 2% agarose gels using iBright imaging system (Thermo Fisher scientific, UK).

### Maternal serum metabolites and hormones

Plasma glucose, leptin, insulin, triglycerides, cholesterol and non-esterified free fatty acids concentrations were determined by immuno and enzymatic assays as described previously by^72^.

### Placental morphological analysis

Fixed placentas were paraffin-embedded and exhaustively sectioned at 7 μm. At least 10 sections representing the entire placental sample were stained with hematoxylin and eosin to determine gross placental structure using superimposed grids and point counting as described previously^36^. Mid-line placental sections stained with hematoxylin and eosin were also used to assess junctional zone morphology as described previously^36^.

Morphology of the labyrinth region was assessed by double-labelling placental sections with rabbit antibodies against cytokeratin (180059, Thermo-Fisher Scientific, UK) and laminin (ab11575, abcam, UK) to identify trophoblast and fetal capillaries, respectively as previously described^73^. Slides were dewaxed, rehydrated and endogenous peroxidase quenched by incubation in 3% (v/v) hydrogen peroxide for 10 min. Sections were subjected to antigen retrieval by microwave heating in citrate buffer (pH 6) for 30 min, washed by three 5 min rinses in phosphate buffered saline (PBS), blocked in 1% bovine serum albumin (BSA) and incubated overnight with anti-laminin antibody (1:200 dilution in 1% BSA/PBS). The following day, goat anti-rabbit secondary antibody (Abcam, ab6720; 1:1000, 1% BSA/PBS) was then added for 1 h followed by streptavidin-horse radish peroxidase complex (Strep-HRP Rockland, S000-03, 1:500 in PBS) for 1 h. Laminin was visualised by staining with diaminobenzidine (DAB; ab64238, Abcam, UK) in saturated ammonium nickel (II) sulphate solution. Sections were then washed, re-blocked in 10% goat serum in 1% BSA and incubated overnight with anti-cytokeratin antibody (1:100 in 10% goat serum and 1% BSA/PBS). Goat anti-rabbit secondary antibody (1:1000, 1% BSA/PBS) was then applied for 1 h, washed and Strep-HRP added. DAB was applied to the sections. Samples were then counterstained with hematoxylin and eosin and mounted in DPX. Eosin helped to identify maternal blood spaces in the labyrinth. Negative control sections were prepared by omission of the primary antibodies. Point counting was used to determine the volume densities of each labyrinth component (fetal capillary, maternal blood space, trophoblast). Labyrinthine fetal capillary length density, total capillary length and diameter were obtained using counting frames. The thickness of the interhemal membrane and surface area were determined using lines and cycloid arcs at random starting locations within the labyrinthine zone. The theoretical diffusion capacity was calculated using the surface area, divided by the interhemal barrier thickness and multiplied by Krogh’s constant for oxygen diffusion (17.3 × 10^−8^ cm^2^ min^−1^ kPa^−1^).

### Placental levels of cell apoptosis

Placental apoptosis was also determined in mid-line placental sections by immuno-labelling with an antibody against cleaved caspase-3 (#9661, cell signalling, MA, USA) using the immunohistochemistry protocol described above. In addition, an *in situ* cell death detection kit, tetramethylrhodamine red (TMR red; Roche, UK) followed by DAPI staining was used to detect the end stage of apoptosis following the manufacturer’s protocol. Cells positive for cleaved caspase-3 or TMR red were counted using ImageJ software and calculated as percentage of total cells.

### Placental gene expression assessment

Gene expression was analysed by real-time PCR (7500 fast real-time PCR system; Applied Biosystems, Cheshire, UK) on RNA isolated from 20 mg of powdered placenta using a RNeasy Plus Mini Kit (Qiagen, Crawley, UK) and reverse-transcribed to cDNA using MultiScribe reverse transcriptase with random primers (Applied Biosystems, Cheshire,UK). Samples were analysed in duplicate using SYBR Green chemistry (MESA Blue qPCR MasterMix, Eurogentec, UK) with gene-specific primers listed in Table 4. The qPCR products were verified by gel electrophoresis and sequencing. The 2^−ΔΔCT^ method for quantification was used for the genes of interest and normalized to the geometric mean expression levels of *L19, Hprt* and *Sdha*, which were unaffected by maternal age.

**Table 4:** PCR primers used for quantification of mRNA expression by real-time RT-PCR with corresponding references, where required.

Transcript	Primers	Reference
Slc2a1	F: 5′-GCTGTGGCTGGCTTCTCTAA-3′	
	R: 5′-CCGGAAGCGATCTCATCGAA-3′	
Slc2a3	F: 5′-ACCTGATTGCCATCCTTGGG-3′	
	R: 5′-AACGATGCCCAGCTGGTTTA-3′	
Slc38a1	F: 5′-CGGCGCCTTTCCCTTTATTTC-3′	
	R: 5′-CCGTTAACTCGAGGCCACTT-3′	
Slc38a2	F: 5′-TTCTGATTGTGGTGATTTGCAAGAA-3′	
	R: 5′-CAGGATGGGCACAGCATACA-3′	
Slc38a4	F: 5′-AAGGTAGAGGCGGGAAAGGG-3′	
	R: 5′-AGGAACTTCTGACTTTCGGCA-3′	
Igf2	F: 5′-GTCGATGTTGGTGCTTCTCA-3′	^74^
	R: 5′-AAGCAGCACTCTTCCACGAT-3′	
Vegf	F: 5′-TCACCGGAAAGACCGATTAAC-3′	
	R: 5′-CCCTTCATGTCAGGCTTTCT-3′	
P53	F: 5′-CCATCCTTACCATCATCACGCTG-3′	
	R: 5′-GGCACAAACACGAACCTCAAAG-3′	
Prl3d1	F: 5′-TTCGGGCTCTGGTATGCAAC-3′	^75^
	R: 5′-TGGACACAATGGCAGTTGGTTTGG-3′	
Prl3b1	F: 5′-ACCATGCTTCTCTGGGACACT-3′	^76^
	R: 5′-AGGCTTCCAGTGGACATTCGGTAA-3′	
Hsd11b1	F: 5′-GAAGAAGCATGGAGGTCAAC-3′	^77^
	R: 5′-GCAATCAGAGGTTGGGTCAT-3′	
Hsd11b2	F: 5′-TGGCCAACTTGCCTAGAGAG-3′	^78^
	R: 5′-TTCAGGAATTGCCCATGC-3’	
L19	F: 5′-CTGAAGGTCAAAGGGAATGTG-3′	^74^
	R: 5′-GGACAGAGTCTTGATGATCTC-3′	
Hprt1	F: 5′-GCCTAAAAGACAGCGGCAAG-3′	
	R: 5′-GGCTGCCTACAGGCTCATAG-3′	
Sdha	F: 5′-TACTGTTGCAGCACAGGGAG-3′	
	R: 5′-TCAGTCCTGCTAAACGGCAT-3′	

### Placental protein abundance analysis

Placentas from 4–5 dams were separated by sex for analysis of protein expression by Western blot. Total protein was extracted using RIPA buffer (Thermo Fisher Scientific, MA, USA) according to manufacturer’s protocol. Lysate protein concentrations were determined using a Bradford assay (Sigma-Aldrich, St. Louis, MO). Equivalent amounts of protein (60 μg) were resolved by SDS-PAGE, blotted onto nitrocellulose (0.2 μm), and analyzed by enhanced chemiluminescence, SuperSignal™ ELISA Femto Substrate (Thermo Fisher Scientific, MA, USA). Intensities of the bands representing IGF2 (ab9574), VEGF (ab46154), 11-βHSD2 (ab80317), GPX1 (ab16798), Catalase (ab1877) (abcam, Cambridge, UK) and Mn-SOD (#06-984, Sigma-Aldrich, Dorset, UK) were determined using iBright analysis software (Thermo Fisher Scientific, MA, USA). Protein loading was controlled for by dividing the measured signal of the bands by that of the Ponceau-S-stained membrane.

### Placental protein oxidation assay

Total protein oxidation levels were detected using OxyBlot^TM^ protein oxidation detection kit (Millipore, Watford, UK) according to manufacturer’s protocol. In brief, proteins were extracted from 60 mg of powdered placenta using lysis buffer. 15 µg of extracted placental protein was then derivatized by 2,4-dinitrophenylhydrazine (DNPH) treatment and separated on 12% SDS-PAGE following electroblotting to nitrocellulose membrane. Membrane bands were visualised using iBright imaging system (Thermo Fisher scientific, Paisley, UK). Proteins were normalised to ponceau-S staining and quantified by iBright analysis software (Thermo Fisher scientific, Paisley, UK).

### Statistical analysis

All data are presented as mean + SEM. Data were normally distributed and analysed by unpaired Student’s t-tests to assess the effect of maternal age within the one fetal sex, using GraphPad Prism 7.00 (GraphPad Software, CA, USA). Data were considered statistically significant at values of p < 0.05.

## Data Availability

All data are available, without restriction, upon request.
