# Author Correction: Advanced maternal age compromises fetal growth and induces sex-specific changes in placental phenotype in rats

**DOI:** 10.1038/s41598-020-59769-8

**Published:** 2020-02-13

**Authors:** Tina Napso, Yin-Po Hung, Sandra T. Davidge, Alison S. Care, Amanda N. Sferruzzi-Perri

**Affiliations:** 1 Centre for Trophoblast Research, Department of Physiology, Development and Neuroscience, University of Cambridge, Cambridge, UK; 2 Department of Obstetrics and Gynaecology, Women and Children’s Health Research Institute, University of Alberta, Edmonton, Alberta Canada; 3 Robinson Research Institute and Adelaide Medical School, University of Adelaide, South Australia, Australia

Correction to: *Scientific Reports* 10.1038/s41598-019-53199-x, published online 28 November 2019

This Article contains an error in Figure 1A-C, where the key has been inadvertently switched. The correct Figure 1 appears below.

**Figure 1:**
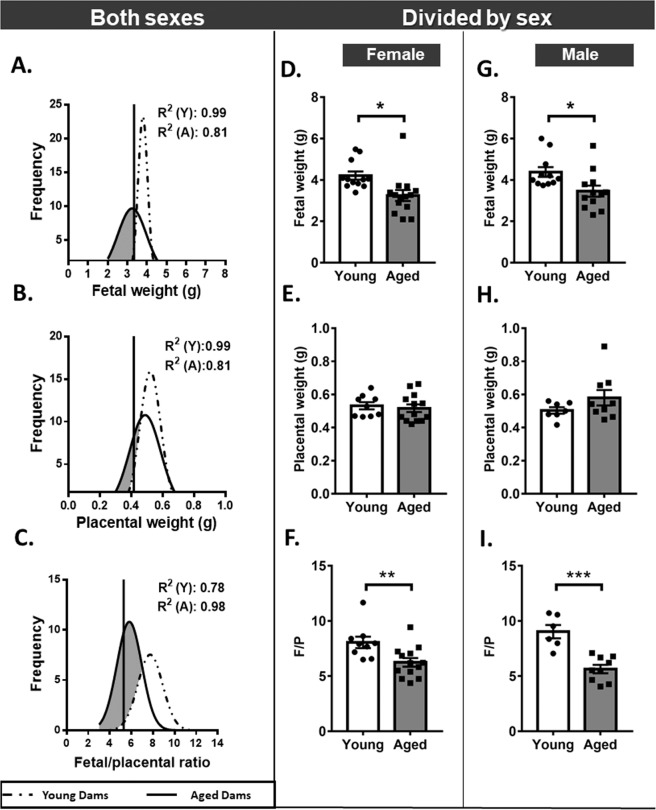
.

